# Delivery of Nucleic Acids and Nanomaterials by Cell-Penetrating Peptides: Opportunities and Challenges

**DOI:** 10.1155/2015/834079

**Published:** 2015-03-26

**Authors:** Yue-Wern Huang, Han-Jung Lee, Larry M. Tolliver, Robert S. Aronstam

**Affiliations:** ^1^Department of Biological Sciences, Missouri University of Science and Technology, Rolla, MO 65409-1120, USA; ^2^Department of Natural Resources and Environmental Studies, National Dong Hwa University, Hualien 97401, Taiwan

## Abstract

Many viral and nonviral systems have been developed to aid delivery of biologically active molecules into cells. Among these, cell-penetrating peptides (CPPs) have received increasing attention in the past two decades for biomedical applications. In this review, we focus on opportunities and challenges associated with CPP delivery of nucleic acids and nanomaterials. We first describe the nature of versatile CPPs and their interactions with various types of cargoes. We then discuss *in vivo* and *in vitro* delivery of nucleic acids and nanomaterials by CPPs. Studies on the mechanisms of cellular entry and limitations in the methods used are detailed.

## 1. Introduction

### 1.1. Cell-Penetrating Peptides

**Figure 1:**
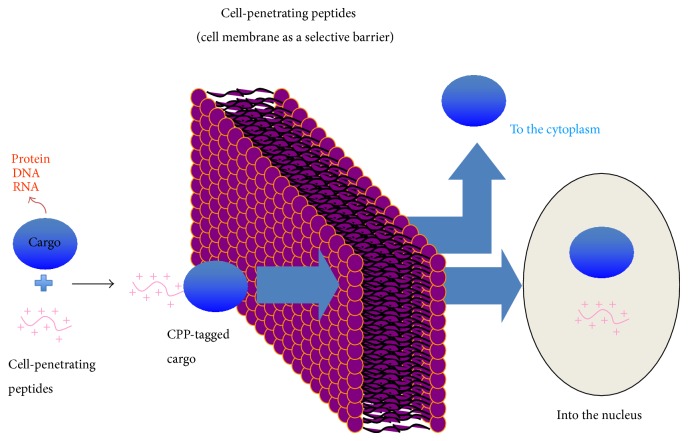
Cell-penetrating peptides as a tool to deliver biologically active molecules.

The plasma membrane plays essential roles in selective permeability, osmotic balance, compartmentalization, and cellular uptake. Small polar molecules such as ions, amino acids, and sugars enter cells through specific carriers and channels in the membrane. Larger macromolecules, such as proteins, DNAs, and RNAs, are generally unable to use this mode of entry. Consequently, delivery tools have been developed to facilitate cellular uptake of large molecules for basic research and biomedical applications (Figure 1). These include mechanical and electrical transfection techniques such as microinjection, bioballistics, hydrodynamic force, ultrasonic nebulization, electroporation, chemical/biochemical methods such as calcium phosphate coprecipitation, membrane fusion catalyzed by artificial lipids, peptides/proteins, dendrimers, adenovirus-associated virus vectors, and lentiviral vectors [1]. Some of these methods are suitable for* in vitro* or* in vivo* use, while others are suitable for both. These delivery methods can also be categorized as involving viral or nonviral carrier systems. Due to safety reasons, nonviral delivery methods such as peptide- and lipid-based systems have received more attention over the past 20 years than viral methods. Advantages of nonviral systems are ease and flexibility of assembly, minimal toxicity, and low levels of immunogenicity and insertional mutagenesis.

Among nonviral delivery methods, cell-penetrating peptides (CPPs) have become increasingly popular. The first CPP was discovered by two independent groups and is comprised of a protein transduction domain (PTD) derived from the transactivator of transcription (Tat) of the human immunodeficiency virus type 1 (HIV-1) [2, 3]. This domain contains eleven amino acids (YGRKKRRQRRR) that are responsible for cellular entry of Tat [4]. Later, a variety of CPPs are derived from natural, chimeric, and synthetic sources (Table 1) [5, 6]. In general, CPPs are (1) less than 30 amino acids, (2) rich in arginine and lysine, (3) positively charged or amphipathic, (4) easy to prepare, and (5) nontoxic [7].

In general, the efficiency of a CPP in mediating cellular uptake is a function of its total electric charge and amino acid sequence insofar as these properties determine its 3-D structures and potential interactions with membranes molecules [8–10]. In particular, secondary amphipathicity is a critical determinant of cellular uptake [9, 11–13]. Data bases and predictive simulation models are available for identifying biomimetic cell-penetrating peptides based upon an array of protein characteristics [14–16].

### 1.2. Versatile CPPs and Their Interactions with Cargoes

**Figure 2:**
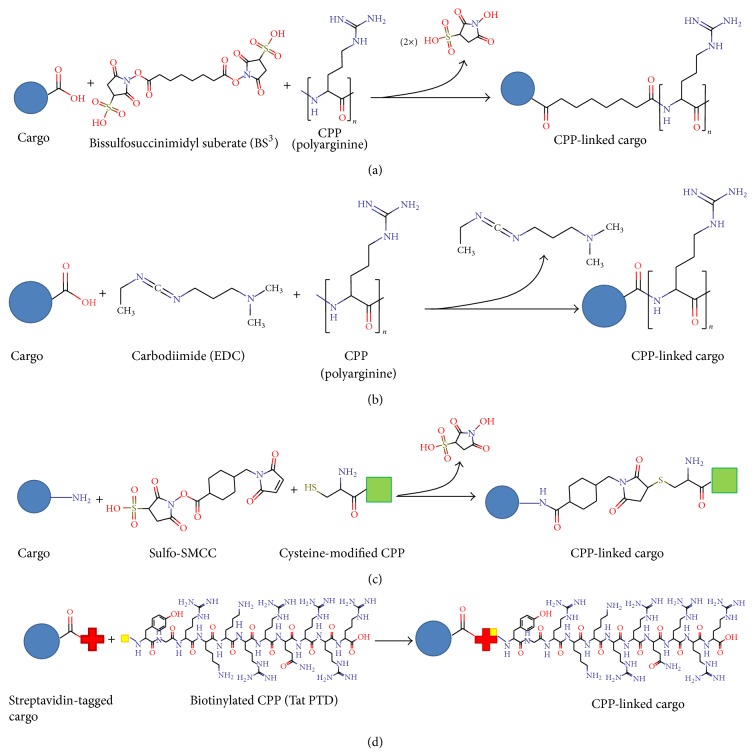
Reaction scheme for linking CPPs to cargoes. The cargoes can be linked to the CPPs through a covalent linkage method such as (a) bissulfosuccinimidyl suberate, (b) carbodiimide, or (c) Sulfo-SMCC with a cysteine-modified CPP, or through a noncovalent method such as (d) biotin-streptavidin interaction.

CPPs have been used as carriers of DNA, RNA, protein, nanomaterials, and pharmaceuticals. Association between CPP and cargo can be either covalent or noncovalent. Covalent interactions have been achieved by sulfosuccinimidyl suberate linkage, carbodiimide conjugation, and thiol-amine coupling. Noncovalent interactions include biotin-streptavidin interactions, electrostatic interactions, and metal-affinity interactions [18–20] (Figure 2). Covalent strategies have been used to conjugate antibody fragments, drugs, and fluorescent labels. Covalent linking ensures strong association between CPP and cargo and high transduction efficiency. However, the covalent-linking procedure may be labor-intensive, time-consuming, and costly. The yield of the CPP-cargo covalent complex also decreases during separation from the unbound CPPs and cargoes. Further, to achieve covalent linking, cargoes are chemically modified, which may compromise functionality. Schwarze et al. first demonstrated the delivery of CPP-fusion proteins into various tissues in mice [21]. Subsequently, others showed that CPPs can carry covalently linked nucleic acids and nanomaterials into cells of a variety cell lines [22–24]. Johnson et al. used the cell-penetrating peptide POD (peptide for ocular delivery) to deliver POD-GFP fusion protein to retina, cornea, and skin [25]. Chang et al. first described a CPP-mediated covalent protein transduction in plants [26].

The advantages of noncovalent binding between CPP and cargo are ease of use, ease of production, versatility with respect to cargo composition, and preservation of cargo functionality [27]. Noncovalent strategies have been used to deliver siRNA, plasmids, and splice correcting oligonucleotides. Noncovalent bonding was applied to the delivery of green fluorescent protein (GFP), collagen and insulin, into mouse skin tissues [28, 29]. We and others have successfully used the noncovalent delivery in several representative organisms of prokaryotes and unicellular yeasts, including cyanobacteria, bacteria, archaea, algae, fungi, and yeasts. However, noncovalent delivery was not successful in multicellular fungi and green algae [30].

## 2. CPPs Delivery of Nucleic Acids

### 2.1. siRNA Delivery

**Figure 3:**
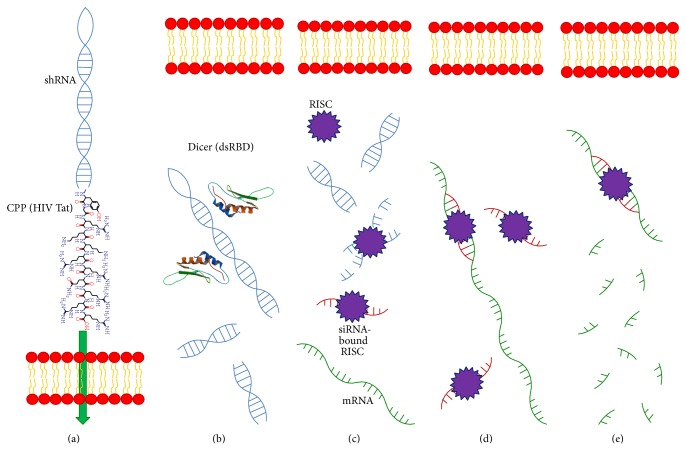
Simplified conceptual diagram (not drawn proportionally in size) of exogenous siRNA-mediated gene silencing. (a) The siRNA (usually small hairpin RNA, shRNA) can be modified to covalently interact with CPPs and then be transported through the cell membrane. (b) shRNA binds to the double-strand RNA binding domain (dsRBD) of the enzyme Dicer and then is processed. (c) The processed RNA is incorporated into the RNA-induced silencing complex (RISC). The passenger strand RNA is degraded. (d) The guide strand RNA along with the RISC binds to a complementary sequence of a targeted mRNA. (e) The targeted mRNA is degraded and translation disrupted.

RNA interference (RNAi) is an evolutionarily conserved mechanism of gene expression regulation in animals and plants [84]. Endogenous pre-microRNAs (pre-miRNAs) are synthesized and processed in the nucleus and then transported to cytoplasm. The pre-miRNA is shortened and processed in the cytoplasm by an RNAse III enzyme (Dicer) to become mature microRNA. A multienzyme complex that includes argonaute 2 and the RNA-induced silencing complex (RISC) binds to the microRNA and eliminates one strand. This activated complex then binds to an mRNA strand that possesses a complementary sequence, thereby inactivating its expression [85–87]. Utilizing this principle, synthetic RNA molecules (small interfering RNA, siRNA) of 20–25 base pairs in length have been developed to manipulate the expression of specific genes (Figure 3). This technique represents a new treatment modality in cancer, infectious diseases, and genetic disorders. Currently there are more than 20 siRNAs undergoing clinical trials in various stages [88]. The biggest challenge of this technique is delivery of siRNA across the cytoplasmic membrane. Carriers are needed to overcome this barrier and CPPs represent an obvious attractive means for siRNA internalization.

The CPPs transportan, penetratin, amphipathic peptides, and polyarginine have been extensively used to covalently or noncovalently deliver siRNA into animal and plant cells [31–33, 36–40, 45, 49, 89, 90]. Target gene products of siRNAs include Luciferase, SOD1, EGFP, p38 MAP kinase, CDK9, VEGF, p53, and Oct-3/4. Stable noncovalent CPP and siRNA complexes can be formed by CPP/siRNA electrostatic interactions. Alternately, CPPs can be covalently linked to siRNA duplexes through disulfide bond formation in which CPPs containing N-terminal cysteines are conjugated to siRNA molecules with a 5′-thiol modified siRNA sense strand [31, 34]. It is critical to purify the CPP/siRNA complex in order to investigate transduction efficiency of the covalent CPP-siRNA complexes. Otherwise, it is difficult to discern the contribution of noncovalent CPP-siRNA complexes to the transduction response.

Potential drawbacks of direct conjugation of cationic CPPs with anionic siRNAs are charge neutralization, inactivation of the CPP, and aggregation/precipitation, which may limit siRNA entry into the cells [33, 91, 92]. Eguchi and Dowdy invented an elegant design that fused a Tat PTD with a double-stranded RNA-binding domain (dsRBD) [93]. This design allowed siRNA to bind to dsRBD while leaving PTD to induce cellular uptake in primary and transformed cells. This tactic was applied in a mouse model of glioblastoma to deliver two siRNAs for simultaneous silencing of EGFR and Akt2. The result was selective destruction of tumor cells and improved longevity of cancerous mice [94]. Clearly, CPP-mediated siRNA delivery has a promising future in disease treatment.

### 2.2. DNA Delivery

The delivery of functional exogenous DNA into organisms is important for transgenic research and gene therapy. Most studies have focused on CPP-mediated delivery into mammalian cells, although our research team has demonstrated that CPPs can deliver biologically active molecules into a variety of species, including rotifers [46], cyanobacteria [95], insects [41], plants [96], and paramecium [42]. Internalization of CPP-mediated DNA transduction involves a combination of pathways including classical endocytosis, caveolin- and clathrin-dependent endocytosis, macropinocytosis, and direct membrane penetration [46, 96, 97]. Various strategies have been developed to enhance transduction efficiency. For instance, stearylation of arginine-rich CPPs drastically increases transduction efficiency of plasmid DNA [35, 98], while hemagglutinin-2 (HA2) analogues or chemicals such as chloroquine and polyethylenimine (PEI) enhance transduction efficiency by catalyzing cargo release from endosomes (see Section 4.3).

There have been attempts to deliver biologically active molecules into the nucleus [99]. Molecules can enter the nucleus from the cytoplasm by either passive diffusion or active transport mechanisms. Small molecules less than 10 nm in diameter or 50–60 kDa in size can diffuse directly through nuclear pore complexes. Most protein molecules are transported by energy-dependent transport mechanisms initiated by nuclear localization signals (NLS). These signals are recognized by importin family proteins that mediate the transport across the nuclear envelope with the participation of Ran proteins [100]. An N-stearylated NLS was found to improve CPP-mediated transfection activity by overcoming cell membrane and nuclear pore barriers [98]. In contrast, we have found that constructs with NLS tag interact with the HA2 sequence thereby limiting delivery. The detailed reason of this NLS interference remained to be elucidated [78].

In addition to penetrating cytoplasmic membrane and nucleus, we demonstrated that a CPP-piggyBac transposase (CPP-PBase) plasmid system could accomplish both protein transduction and transposition [101]. The system was able to synchronously deliver covalently linked PBase and noncovalently linked cis plasmid into human cells. This one-plasmid “transposoduction” has tremendous potential for safe and efficient cell line transformation, gene therapy, and functional genomics.

## 3. CPPs Delivery of Nanomaterials

The improved sensitivity, resolution, and versatility of fluorescent microscopy and the discovery of fluorescent proteins have revolutionized imaging in basic science and biomedical applications [102, 103]. These fluorescent proteins have been extensively used for visualizing and tracking molecules in dynamic cellular processes. They may also be useful in disease diagnosis and therapeutic planning. Recently Nguyen et al. advanced the possibility of utilizing fluorescent proteins to improve surgical precision [104]. However, the broad emission spectra of current organic fluorophores impede multiplex imaging, while photobleaching limits their use in long-term imaging [105, 106]. Furthermore, cell autofluorescence in the visible spectrum and a need of probes that emit in the near-infrared (NIR) region drive the need to develop new imaging probes.

**Figure 4:**
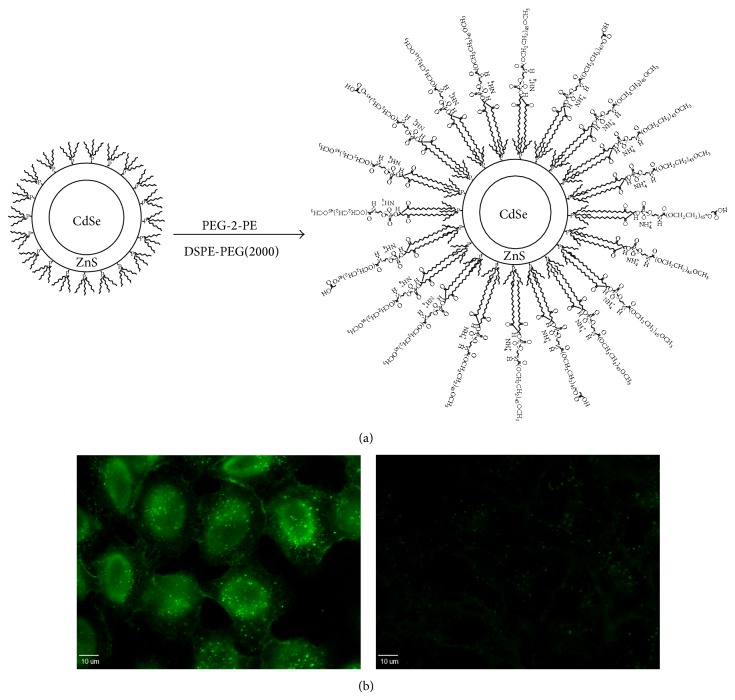
(a) Synthesis of water-soluble carboxylated CdSe/ZnS quantum dots. Upon addition of ZnS as a shell to protect Cd core, the surface was modified with 1, 2-distearoyl-sn-glycero-3-phosphoethanolamine-N-[carboxy(polyethyleneglycol)-2000] (DSPE-PEG 2000) and 1,2-dipalmitoyl-sn-glycero-3-phosphoethanolamine-N-[methoxy(polyethyleneglycol)-2000] (PEG-2 PE). The amount and ratio of PEG2-PE and DSPE-PEG(200) determine suspension stability in water. (b) Fluorescence of CdSe/ZnS quantum dot in live cells with (left) and without (right) nona-arginine after a 1-hour exposure [17].

Nanomaterials are materials that have at least one dimension in the range of 1–100 nm. The development of nanomaterials has revolutionized many industries such as computing and semiconductor, optics, energy, and cosmetics [107]. Semiconductor nanocrystals (a.k.a. quantum dots, QDs) possess high optical extinction coefficient, a narrow range of emission wavelength, exceptional resistance to photo- and chemical degradation, and high quantum yield [108, 109]. These properties make QDs particularly attractive for long-term observation of molecules in live cells and multiplex imaging, as well as tumor targeting and diagnostics* in vivo*. However, inorganic QDs are not permeable to cytoplasmic membrane and agglomerate easily. Thus, surface modifications of QDs, such as complexing with polyethylene glycol, are required to achieve stable suspension (Figure 4(a)). Even so, QDs are poorly taken up by cells (Figure 4(b)). Josephson et al. first reported increased uptake of iron oxide nanoparticles covalently conjugated with Tat-PTD [110]. These Tat-iron oxide nanoparticle complexes were internalized into lymphocytes and yielded magnetic labeling of cells. This technology opens up the possibility for simultaneous diagnosis and treatment of diseases (i.e., theranostics) when drugs are included in the imaging system.

Stroh et al. successfully labeled primary bone marrow cells with Tat-QD micelles* ex vivo* and observed the recruitment of the labeled bone marrow-derived precursor cells to the tumor vasculature [111]. This methodology may advance our understanding of stem cell proliferation and differentiation. Many other studies have investigated CPP-mediated delivery of QDs into living cells for basic science and biomedical application purposes [17, 24, 43, 44, 46–48, 112–117]. In general, cellular uptake of the CPP/QD complexes includes classical endocytosis, macropinocytosis, and direct membrane penetration. Factors that influence uptake efficiency of CPP/QD complexes include the size and the overall surface charge of the complexes. For instance, our data suggest that electropositive charges of CPP/QD complexes (measured as zeta-potential) increase higher transduction efficiency [118].

**Figure 5:**
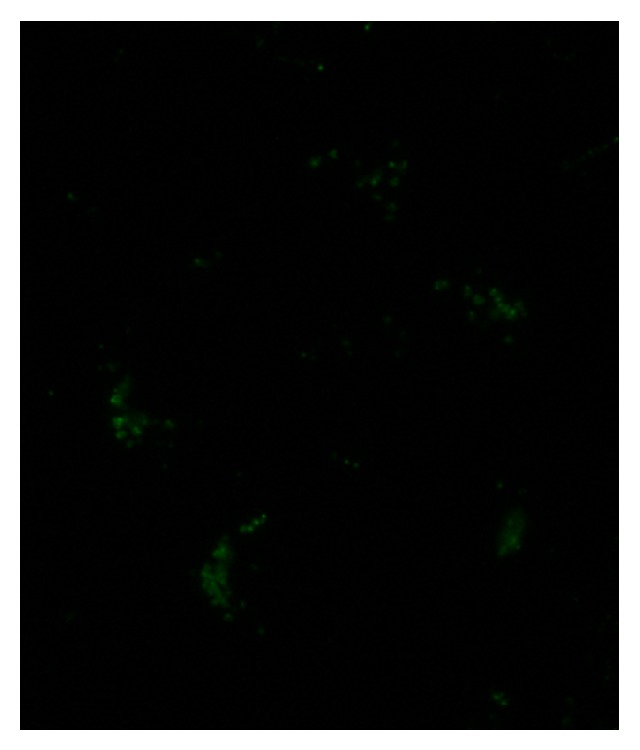
HR9 CPP facilitates cellular uptake of green fluorescent nanodiamonds.

Although Cd-based QDs at nontoxic levels can be useful in research applications [119], they are not ideal agents for therapeutic purposes. Biocompatible, fluorescent nanodiamonds represent an attractive alternative. Defect center (color center) of nanodiamonds can be created by irradiation with a high power laser beam followed by thermal annealing at 800°C [120]. Nanodiamonds thus have been modified producing strong and stable fluorescence with no photoblinking (within 1 ms) and no photobleaching [121]. We found that histidine-modified arginine-rich CPP (HR9) can facilitate the cellular uptake of these fluorescent nanodiamonds (Figure 5). Collectively, a combined use of CPPs and nanoscaled materials (with or without fluorescence) may greatly enhance payload and efficiency for imaging and therapeutic uses.

## 4. Mechanisms of CPP-Mediated Cellular Uptake

### 4.1. Complementary Methods to Study Mechanisms of Cellular Uptake

**Figure 6:**
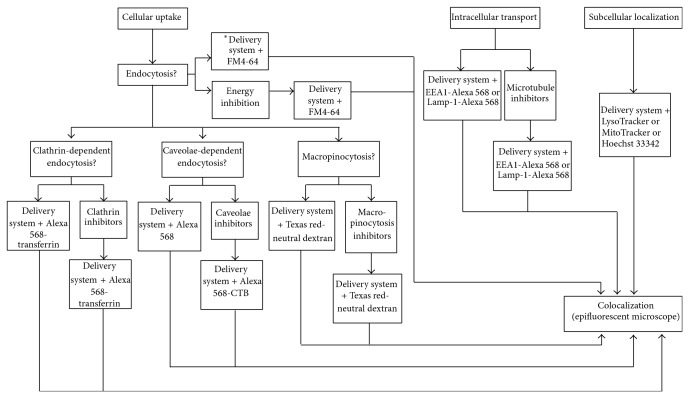
Diagram illustrating a comprehensive workflow of experiments designed to characterize the cellular uptake, intracellular uptake, and subcellular localization of CPPs and their cargoes.

**Figure 7:**
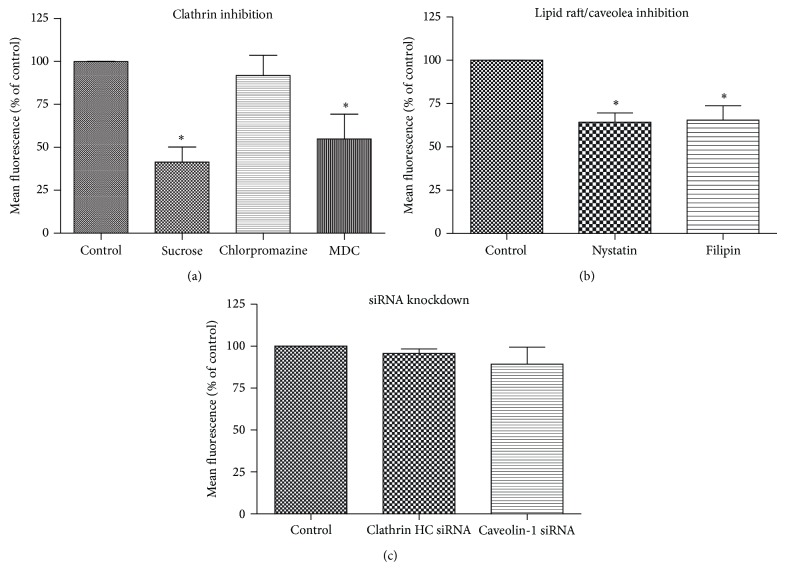
Comparisons of clathrin- and caveolin-dependent cellular uptake pathways using pharmacological inhibitors and RNAi technique [17].

Fixed cell imaging was utilized to study CPP-mediated cellular uptake. However, the fixation procedure introduced artifacts and yielded inaccurate data. Live cell imaging has supplanted fixed cell imaging and become a powerful tool to study dynamic cellular process in CPP-mediated uptake. Coupled with cellular uptake markers and organelle markers, the subcellular localization of the CPP/cargo complex can be identified (Table 2).  Figure 6 presents a comprehensive workflow of experiments on cellular uptake, intracellular uptake, and subcellular localization. There are some discrepancies among publications regarding the identity of CPP uptake mechanisms due to the limited use of cellular process inhibitors. Although pharmacological inhibitors (Table 3) can be used to inhibit internalization processes, these inhibitors are not completely specific and may suppress more than one cellular uptake pathway. For instance, cytochalasin D (CytoD) and* N*-ethylmaleimide (NEM) inhibit both clathrin- and caveolin-mediated pathways. CytoD also inhibits macropinocytosis. This makes it difficult to evaluate the contributions of different pathways to transduction and complicates the analysis of CPP-mediated uptake mechanisms. To overcome this problem, we suggested that RNAi be used as a complementary method to thoroughly elucidate CPP-mediated uptake mechanism. For instance, in our study with CPP-mediated cellular uptake of CdSe/ZnS quantum dots, pharmacological inhibitors reduced cellular uptake of the noncovalent CPP/QD complex. However, uptake efficiency of the CPP/QD complex was not reduced by siRNAs introduced to knockdown clathrin HC and caveolin 1 (Figure 7). It is also worth noting that although the effective dose of an inhibitor may be specified by commercial vendors or literature, a pilot study should be conducted to optimize the concentration of an inhibitor for a specific cell line since too high concentration of an inhibitor may be toxic to cells and compromise cellular processes.

### 4.2. Diverse Cellular Uptake Routes

Understanding the mechanisms underlying CPP-mediated cellular uptake and subcellular localization of the carrier system is needed to improve transduction efficiency and cargo functionality. Our understanding of uptake is still incomplete. Proposed routes of entry include direct membrane penetration and various types of endocytic pathways. Empirical modeling evidence from several studies supports a direct membrane penetration. Initially CPPs bind to the phosphate groups of the phospholipids on the bilayer surface. As the concentrations of CPPs on cell surface increase, the lipid molecules rearrange. Side chains of arginines translocate through the distal layer and form a water pore. Finally, a few CPPs diffuse through the pore, followed by pore closure [122–125].

Most studies of CPP-mediated cellular uptake of nucleic acids and QDs have focused on endocytosis. Endocytosis is an active process whereby cells internalize extracellular material through cytoplasmic membranes. This process is required by certain cells to obtain essential nutrition and excrete cellular waste. At least 10 different types of endocytic pathways involving various molecules have been delineated [126]. Studies of cellular uptake of CPP/cargo complexes have focused on three pathways: clathrin-mediated endocytosis, caveolin-dependent endocytosis, and macropinocytosis [17, 34, 43, 44, 97, 127, 128]. Future studies should consider other endocytic pathways: CLIC/GEEC, IL2R*β*, Arf6-dependent, flotillin-dependent, circular dorsal ruffles, and entosis.

In endocytosis, CPP/cargo complexes might initially interact with heparan sulfate proteoglycans (a pool of anionic charge on the cell surface). However, Gump et al. recently revised the role of glycosaminoglycans in Tat PTD-mediated induction of macropinocytosis [129]. They found that transduction occurs efficiently in the absence of glycosaminoglycans and sialic acid and that the removal of cell surface proteins totally abolishes transduction. They suggested that additional cell surface protein(s) are necessary for Tat PTD transduction. More studies are needed to identify these proteins are and appreciate their roles in CPP membrane transduction.

Collectively, current data suggest that the routes of the cellular uptake for CPP/cargo complexes are diverse, reflecting the varied chemical and physical natures of the CPPs and cargoes: entry may simultaneously involve multiple routes.

### 4.3. Release from Lysosomal Entrapment

A particular problem associated with most of the CPP delivery systems is entrapment in lysosome, which may lead to cargo degradation and, thus, loss of intended functionality. Multiple strategies have been developed to circumvent this problem. One method is to add to the CPP a section of the hemagglutinin (HA) sequence from the human influenza virus (Table 4). HA is composed of two subunits: hemagglutinin-1 responsible for binding to cells and hemagglutinin-2 (HA2) responsible for lysosomal escape [130]. The N-terminal domain of the HA2 subunit possesses 23 amino acids in a hydrophobic region referred to as fusion peptide [131]. This fusion peptide domain is buried inside the HA trimer in its resting conformation. Upon acidification in the lysosome, an irreversible conformational change of HA2 occurs, exposing the fusion peptide and allowing it to insert into lysosomal membranes. Subsequently a fusion pore is in the membrane is formed, leading to transport of lysosomal contents into the cytosol.

The sequence of CPP-HA2 can be chemically synthesized or the HA2 sequence can be inserted into a CPP-containing plasmid. The advantages of using a peptide synthesizer to produce a CPP-HA2 sequence are high purity, ease of programming the sequence, and flexibility of residue modification as well as molecular conjugation. Disadvantages include limited length of the sequence, loss of yield during purification process, and possible loss of native configuration. The advantages of using a CPP-HA2 plasmid are low cost, time saving for production, and the flexibility to include other desired functional sequences such as imaging molecules. Disadvantages are low purity and considerable time and labor investments. In addition to HA2 and its analogues, the sequences of CPPs can be modified for lysosomal escape. Collectively, these HA2 analogues and sequence variations of CPPs exhibit different degrees of enhanced transduction efficiency, ranging from 0.2- to 7000-fold (Table 4). Factors influencing efficiency include the nature of CPPs and HA2, types of cargoes, and sequence orientation of CPP and HA2.

In addition to fusogenic amino acid sequences, chemicals such as chloroquine and polycationic polyethylenimine (PEI) are commonly used to promote lysosomal escape. Chloroquine, a weak base, can enter the cell and accumulate in vesicular compartments following protonation. At low concentrations, chloroquine inhibits endosome acidification and maturation by preventing the accumulation of free protons. As its concentration increases, it starts to accumulate counterions to protons (e.g., chloride ion) in endosomes, leading to endosomal swelling and rupture [132, 133]. Endosomal release by chloroquine enhances of transduction efficiency [78, 134, 135]. The secondary and tertiary amines of low molecular weight PEI can be protonated in the acidic environment of the endosome, leading to endosomal swelling and rupture. PEI has been used to deliver DNA plasmids with improved transduction efficiency [81, 136–139]. The drawback of the PEI polymer is that it is not biodegradable and is highly charged. Thus interaction of this polymer with genetic materials in the cell nucleus might alter gene expression [140–142].

## 5. Conclusions

CPPs are capable of carrying nucleic acids and nanomaterials into cells. CPPs can interact with cargoes in covalent or noncovalent manners. Complementary tools such as pharmacological inhibitors and siRNA are being used to decipher mechanisms of cellular uptake. Depending on the physiochemical natures of the CPP/cargo complex, the mechanism of cellular entry may include classical endocytosis, macropinocytosis, clathrin- and caveolon-dependent pathways, and direct membrane penetration. A variety of chemical and molecular methods have been introduced to overcome lysosomal entrapment in order to achieve higher functional yields. As studies continue to advance our understanding about CPPs, this delivery modality will find considerable usage in clinical setting and basic science research.

## Figures and Tables

**Table 1:** A variety of cell-penetrating peptides mentioned in this paper.

CPP	Amino acid sequence	References
Viral or natural CPPs		
HIV Tat	YGRKKRRQRRR	[31–35]
HIV Rev	TRQARRNRRRRWRERQR	[35]
FHV coat	RRRRNRTRRNRRRVR	[35]
HSV-1 protein VP22	DAATATRGRSAASRPTERPRAPARSASRPRRPVD	[34]
Penetratin	RQIKIWFQNRRMKWKK	[31, 33, 36, 37]
EB1 (penetratin analog)	LIRLWSHLIHIWFQNRRLKWKKK	[31]
MPG	GALFLGFLGAAGSTMGAWSQPKKKRKV	[31, 34, 38–40]
Polyarginines		
PR9	FFLIPKGRRRRRRRRR	[41–44]
SR9	RRRRRRRRR	[31, 34, 41, 42, 45]
IR9	GLFEAIEGFIENGWEGMIDGWYGRRRRRRRRR	[46–48]
HR9	CHHHHHRRRRRRRRRHHHHHC	[41–44, 46]
Engineered CPPs		
Transportan	CLIKKALAALAKLNIKLLYGASNLTWG	[31, 36]
CADY	GLWRALWRLLRSLWRLLWRA	[31, 49]
C6	RLLRLLLRLWRRLLRLLR	[13]
C6M1	RLWRLLWRLWRRLWRLLR	[13]
PF20 (and variants, see [9])	LLKLLKKLLKLLKKLLKLL	[9]
NAP	KALKLKLALALLAKLKLA	[9]
Steryl-NAP	Stearyl-KALKLKLALALLAKLKLA	[9]
POD	GGG[ARKKAAKA]_4_	[25]

**Table 2:** Examples of cellular uptake markers and organelle markers for green fluorescent CdSe/ZnS quantum dots in live cell imaging.

Marker	Function	Color (Ex/Em)	Incubation time	Conc.
FM4-64	Endocytosis marker	Red (506/750)	15 min	2 *μ*M
Alexa Fluor 568-Transferrin	Clathrin-dependent endocytosis marker	Red (580/630)	5 min	25 *μ*g/mL
Alexa Fluor 568-Cholera toxin B	Caveolae-dependent endocytosis marker	Red (580/630)	10 min	5 *μ*g/mL
Texas red-Neural Dextran 70	Macropinocytosis marker	Red (595/615)	30 min	5 *μ*M
TMR-Dextran	Early endosome marker	Red (555/580)	5 min	10 mg/mL
Lyso Tracker Red DND 99	Lysosome marker	Red (577/590)	10 min	0.5 *μ*M
EEA1-Alexa Fluor 568	Early endosome marker	Red (580/630)	5 min	10 *μ*g/mL
Lamp-1-Alexa Fluor 568	Late endosome marker	Red (578/603)	5 min	10 *μ*g/mL
Mito Tracker Deep 633	Mitochondria marker	Red (640/662)	10 min	1 *μ*M
Hoechst 33342	Nuclei marker	Blue (352/461)	30 min	5 *μ*M

**Table 3:** Pharmacological and physical inhibitors of cellular uptake process.

Inhibitor	Mechanism	Working condition	Ref.
Low temperature	Inhibit energy-dependent endocytosis	4°C	[17]
Hypertonic medium	Inhibit clathrin-dependent endocytosis (Dissociate clathrin lattice)	0.2–0.45 M sucrose	[17, 50]
Potassium depletion	Inhibit clathrin-dependent endocytosis (Dissociate clathrin lattice)	50% DMEM w/1 mM ouabain	[50]
Fusicoccin	Endocytosis inhibitor (H^+^-ATPase activator)	10 *μ*M	[51]
Valinomycin/nigericin	Inhibit energy-dependent endocytosis (Na^+^/K^+^ ATPase modulator; K^+^ selective ionophore)	2 *μ*M	[52, 53]
Sodium azide/sodium fluoride/antimycin A	Inhibit energy-dependent endocytosis (All metabolic inhibitors)	(0.15%/15 mM/2 *μ*g/mL)	[17]
Okadaic acid	Endocytosis and autophagy inhibitor	1.5 *μ*M	[4, 26]
Nocodazole	Inhibit clathrin-dependent endocytosis (Cause microtubule depolymerization)	10–25 *μ*M	[17, 54]
Latrunculin A	Inhibit micropinocytosis (F-actin depolymerization inhibitor)	15 *μ*M	[55]
Cytochalasin D (CytD)	Inhibit macropinocytosis, clathrin- and caveolae-dependent endocytosis (Inhibit F-actin rearrangement)	1–30 *μ*M	[56–58]
*N*-Ethylmaleimide (NEM)	Inhibit clathrin- and caveolin-dependent endocytosis (Inhibit endosomal fusion, energy metabolism)	0.1–3 mM	[59–61]
Methyl-*β*-cyclodetritrin (M*β*CD)	Inhibit clathrin- and caveolae-dependent endocytosis (Deplete or sequester cholesterol)	2 mM	[62]
Filipin	Inhibit caveolae-dependent endocytosis (Inhibit lipid raft; cholesterol binding)	5 *μ*g/mL	[63]
Nystatin	Inhibit caveolae-dependent endocytosis (Inhibit lipid raft; sequester cholesterol)	5 *μ*g/mL	[63]
Wortmannin	Inhibit receptor-mediated endocytosis (Inhibit PI-3 kinase)	100 nM	[64]
5-(N-Ethyl-N-isopropyl)-amiloride (EIPA)	Inhibit micropinocytosis (Inhibit Na^+^/H^+^ exchanger)	100 *μ*M	[65]
Dextran sulfate	Inhibit binding of CPP to cell membrane	5 *μ*g/mL	[66]
DMSO/ethanol	Direct membrane translocation enhancer	10%/1%	[43]
Oleic acid/limonene/PEG	All direct membrane translocation enhancers	5%	[28, 43]
Chloroquine	Lysosomotropic agent (Cause vesicular lysis)	100 & 25 *μ*M	[67, 68]
Ammonium chloride	Inhibit the fusion of lysosomes with endosomes	10 mM	[67, 69]

**Table 4:** HA2 analogues and other sequence variations in CPPs to overcome lysosomal entrapment.

Name	Amino acid sequence	a.a. #	Structural order	Cargo	Purity	Efficiency	Ref.
HA2 analogues							
	GLFEAIEGFIENGWEGMIDGWYG	23	pHA2-p53-R9	p53	N.A.	5x	[70]

	GDIMGEWGNEIFGAIAGFLG	20	pTat-Cre, pTat-HA2	No cargo	N.A.	2–6x	[71]

	GLFEAIEGFIENGWEGMIDGWYG	23	HA2-Tat	PM10	N.A.	0.5–1x	[72]

	pTat-HA-hARC or pTat-HA-*β*-gal		pTat-HA-hARC or pTat-HA-*β*-gal	*β*-gal, ARC	N.A.	N.A.	[73]

	GLFEAIAEFIEGGWEGLIEGCAKKK	25	HA2-NT (NT = neurotensin)	NT	>90%	22%	[74]

	GLFGAIAGFIENGQWGMIDG	20	HA2-Tat	FP	N.A.	N.A.	[75]

	GLFEAIEGFIENGWEGMIDGWYG	23	HA2-Tat	Shepherdin	>90%	3x	[76]

	GLFEAIEGFIENGWEGMIDGWYGC GLFEAIEGFIENGWEGMIDGWYGC (dimeric)	48	EGFR siRNA/LF/diNF-7	EGFR siRNA	N.A.	>2x	[77]

	GLFEAIEGFIENGWEGMIDGWYG	23	CPP-HA2	mCherry	N.A.	80–90x	[78]

	GLFEAIEGFIENGWEGMIDGWYG	23	GALA-INF	Luciferase	N.A.	>1000x	[79]

	GLLEALAELLE	11	HA2-CPP	FP	>95%	2x	[80]

Sequence variations in CPPs							
10H	CHHHHHRKKRRQRRRRHHHHHC	22	C-5H-Tat-5H-C	Luciferase	>98%	>7000x	[81]

HR9	CHHHHHRRRRRRRRRHHHHHC	21	C-5H-R9-5H-C	DNAs, FPs, QDs	87%	20x	[43]

PasR8	FFLIPKGRRRRRRRRGC	17	Pas-CPP	Alexa 488	N.A.	18x	[82]

PR9	FFLIPKGRRRRRRRRR	16	Pas-CPP	DNAs, FPs, QDs	99%	47.5x	[44]
GALA	WEAALAEALAEALAEHLAEALAEALEALAA	30	GALA-EGFP	FITC	N.A.	68x	[83]

N.A. = not available.

## Abbreviations

CPP: Cell-penetrating peptide
CDK9: Cyclin-dependent kinase 9
dsRBD: Double-stranded RNA binding domain
GFP: Green fluorescent protein
EGFP: Enhanced green fluorescent protein
HA: Hemagglutinin
MAP kinase: Mitogen-activated protein kinase
NLS: Nuclear Localization Signal
POD: Peptide for ocular delivery
PTD: Protein transduction domain
PEI: Polyethylenimine
QD: Quantum dot
RISC: RNA-induced silencing complex
RNAi: RNA interference
RNAse: Ribonuclease
siRNA: Small interfering RNA
SOD1: Superoxide dismutase 1
VEGF: Vascular endothelial growth factor.
